# A Pining

**DOI:** 10.4103/0019-5545.39772

**Published:** 2008

**Authors:** Fr. Kurien George

**Affiliations:** *Fr. Kurien George, the director of a halfway home attached to the Mar Thoma Church, in Pathanamthitta District, Kerala, completed his Master's degree in Psychology from City University of New Youk, USA. Fr. George has also trained in Family Counseling with the Princeton Theological Seminary, Prinecton, New Jersey. He has published a book of poems entitled, “Echoes of Life” in 1996. He can be contacted at kgachen@hotmail.com. The above poem was sent by Dr. P.K. Kuruvilla, Psychiatrist from the same district.*

Unlock Unlock the fettered bonds

That binds the heart and seals the sound

Waves and bellows crowd my soul

In my pain, I am alone.

I wait for ‘open sesame’, soul's release

I seek freedom... a shattering unease

Why should I be bound like this?

Locked in and out, no beauty or bliss.

Yet, in my depth I see... freedom

And

As I try to grasp its slithering form

Slipping away into crevices deep

Hiding from my faith-filled leap

I want this freedom but how do I make it mine?

An answer... For this I pine.

